# Comparison of closure versus non-closure of the intraoral buccal mucosa graft site in urethroplasties. A systematic review and meta-analysis

**DOI:** 10.1080/2090598X.2022.2097613

**Published:** 2022-07-18

**Authors:** Yavuz Güler

**Affiliations:** İstanbul Rumeli University, Private Safa Hospital, Urology Department, İstanbul, Turkey

**Keywords:** Buccal graft, closure, non-closure, urethroplasty, urethral stricture

## Abstract

**Aim:**

To assess postoperative oral morbidity through meta-analysis of comparative studies for closure or non-closure of the buccal mucosa graft harvest area in patients undergoing urethroplasty.

**Methods:**

A systematic literature review was conducted in January 2022. Randomized controlled studies were assessed according to the Cochrane collaboration guidelines. Postoperative pain, difficult mouth opening, alteration of oral salivation, perioral numbness, and tolerance of solid and liquid intake results were assessed. Standard mean differences and risk ratios with 95% confidence intervals were estimated for relative risk. Assessment was performed with subgroup analyses according to time points.

**Results:**

This meta-analysis included 373 patients in 7 randomized studies. The oral pain overall pooled effect estimates were investigated for the time points of day 0–1, day 3–7 and months 1–6. According to corrected effect estimates after sensitivity analysis, at the day 0–1 time point, the non-closure group was significantly superior compared to the closure group. But there was no difference at the other time points and in total. The overall pooled effect estimates for difficult mouth opening were investigated at 4 time points (day 1, days 5–7, months 1–3 and months 6). After sensitivity analysis, the overall pooled effect estimates at 6 months were significantly superior for the non-closure group. There were no significant differences between the non-closed and closed groups based on the overall pooled-effect estimates for oral numbness, salivary secretion alteration, and tolerance of liquid and solid food variants.

**Conclusion:**

The non-closure group was more advantageous in terms of oral pain in the early postoperative period. There were no differences between the groups in terms of alteration of salivation, oral numbness and toleration of liquid/solid food. Although the non-closed group seems more advantageous in terms of ease in mouth movements, more studies are needed to prove this.

## Introduction

Urethral stricture is a frequently observed disease, especially in elderly men [1]. The main etiology is blamed on infections, idiopathic causes, trauma and previous urethral operations [2]. For treatment of urethral stricture, optical urethrotomy interni is the most commonly used method. However, urethral stricture disease may recur, although this is more common with infectious causes, complicated and long urethral strictures [3]. The gold standard surgery for treatment of recurrent stricture is urethroplasty [4]. The male urethral stricture guidelines of the American Urological Association (AUA) recommend urethroplasty for recurrent and complicated stricture of the meatus and fossa, all stricture in the penile urethra and recurrent strictures longer than > 2 cm in the bulbar urethra [5].

The use of buccal mucosa as graft in urethroplasty dates back to the 1890s [5]. The first case series was presented in the study in 1993 by El-Kassaby et al. [6]. Later from the end of the 90s and beginning of the 2000s urethral stricture reconstruction surgery began to be performed more often [7–11]. Due to advantages like the ability to easily obtain buccal mucosa, lack of hair follicles, having lamina propria rich in fine veins, easy inosculation and imbibition into the urethral bed, and adaptation to the wet environment, buccal mucosa is a successful graft source in the long term for urethral stricture [12].

Oral mucosa graft operation is a method with low morbidity performed with 98.2% patient satisfaction [13]. Though not serious, some complications may occur in the early and late postoperative periods in the buccal mucosa harvest field in the oral cavity. Probable complications include intraoperative bleeding, infection, pain, swelling, difficult mouth opening, oral numbness, salivation problems, and parotid gland canal injury [14,15].

Closure or non-closure of the buccal mucosa defect area in the oral cavity has been debated for a long time [16]. The advantages of primary suturing include bleeding control and more rapid healing of the wound. Contrary to this, leaving the wound open, or secondary healing, is proposed to be more advantageous for oral pain and difficult mouth opening. For this reason, the topic of whether to perform primary suturing of the defect in the oral cavity or leave it for secondary healing to reduce these perioperative and postoperative complications and to increase patient quality of life and satisfaction with surgery is still controversial. In this meta-analysis, we assessed whether or not closure or non-closure of the donor field in urethroplasty patients with buccal graft replacement for male urethral stricture induced a difference in postoperative oral morbidity.

## Materials and methods

### Data sources and search strategy

A systematic literature search was performed in January 2022 in the following electronic databases: PubMed, Embase, Scopus, Cochrane Central Register of Controlled Trials, Web of Science, Global Index Medicus, ClinicalTrials.gov and Google scholar. We also searched the references of full articles retrieved in our review to identify any additional studies.

No language restrictions were applied in the literature review. An extensive and comprehensive search was undertaken for appropriate studies to minimize reporting bias, publication bias, and their potential impact.

These arch terms used to identify potentially eligible studies from each data source were as follows: ‘buccal graft’; ‘closure’; ‘non-closure’; ‘urethroplasty’; and ‘urethral stricture’. We also scanned the reference lists of related studies. One reviewers independently identified, screened and evaluated the citations and abstracts in the scientific literature related to buccal mucosa graft (BMG) harvesting site management in both adult and pediatric populations. This systematic review considered all comparative studies that assessed patient-reported outcomes and morbidities related to non-closure vs. closure of BMG harvest sites. The articles that either reviewer flagged were retrieved for further full-text evaluation. Articles were taken for further full-text review. Each full-text article was then reviewed and determined whether the inclusion criteria were met. When a study has more than one publication, only the most recent complete data reporting was considered to extract further data.

The identified randomized controlled trials (RCTs) that compared non-closure vs. closure (as control) of the BMG harvest site were appraised using the risk of bias tool according to the Cochrane Collaboration recommendations for intervention reviews [11]. The outcomes of the RCTs were then extracted and included in the meta-analysis.

### Assessment of study quality

The methodological quality of RCTs was evaluated using Cochrane collaboration tools, including 6 items: randomization reviewers, allocation concealment, blinding of personnel and participants, blinding of outcome measurement, incomplete outcomes, selective reporting and other bias [17]. This study followed the phrase PRISMA (preferred reporting items for systematic reviews and meta-analyses) [18].

### Data extraction, inclusion and exclusion criteria

This meta-analysis included patients with urethral stricture over 18 years of age, with unilateral in-cheek buccal mucosa graft harvest performed, comparing closure and non-closure of the donor field. Studies performed with adolescents, women and children were excluded. Details about the study characteristics and primary outcome assessments were extracted and tabulated by 1 reviewer and counter verified by another. The details of studies included in this meta-analysis are summarized in the table. The primary outcomes of this meta-analysis were difficult mouth opening (time measurement point up to 6 months), oral numbness (time measurement point up to 12 months), oral pain score (assessed with visual analogue scale or numerical rating scale, time measurement point up to 6 months), alteration of salivation or salivatory problems (time measurement point up to 6 months), toleration of liquid (time measurement point up to 7 days) and toleration of solid diet (time measurement point up to 6 months).

### Data synthesis and analysis

The Review Manager 5.3 software (Cochrane Collaboration, Oxford, UK) statistical package was used to perform the statistical analyses. The standardized mean difference (SMD) with corresponding 95% confidence intervals(CIs) was generated for between-group treatment effect estimations. The SMD refers to the effect size of the intervention relative to the variability observed in each individual study; therefore, it is considered more generalizable and appropriate to standardize the results of different studies that evaluate the same outcome but measure it with different validated scales.When the event rate of oral morbidity was described by the included study, it was extrapolated as dichotomous data and pooled as relative risk (RRs) with corresponding 95% CIs. The proportion of heterogeneity across the studies was tested using the *I* ^2^index (range: 0%–100%). If *I ^2^ *< 50%, the variation of the studies was considered to be homogenous and the fixed-effect model wa sadopted.

If *I^2^ *> 50%, the variation of the studies was considered to be significantly heterogeneous and the random-effect model was adopted. All P values were two-tailed, and p < 0.05 was considered statistically significant. Sensitivity analysis was performed to detect studies causing heterogeneity. The corrected overall pooled effect estimate values were extracted for these studies.

### Publication bias

Potential publication bias was evaluated by identifying visual asymmetry/symmetry on funnel plot studies.

## Results

### Search results and characteristics of included studies

**Figure 1.**
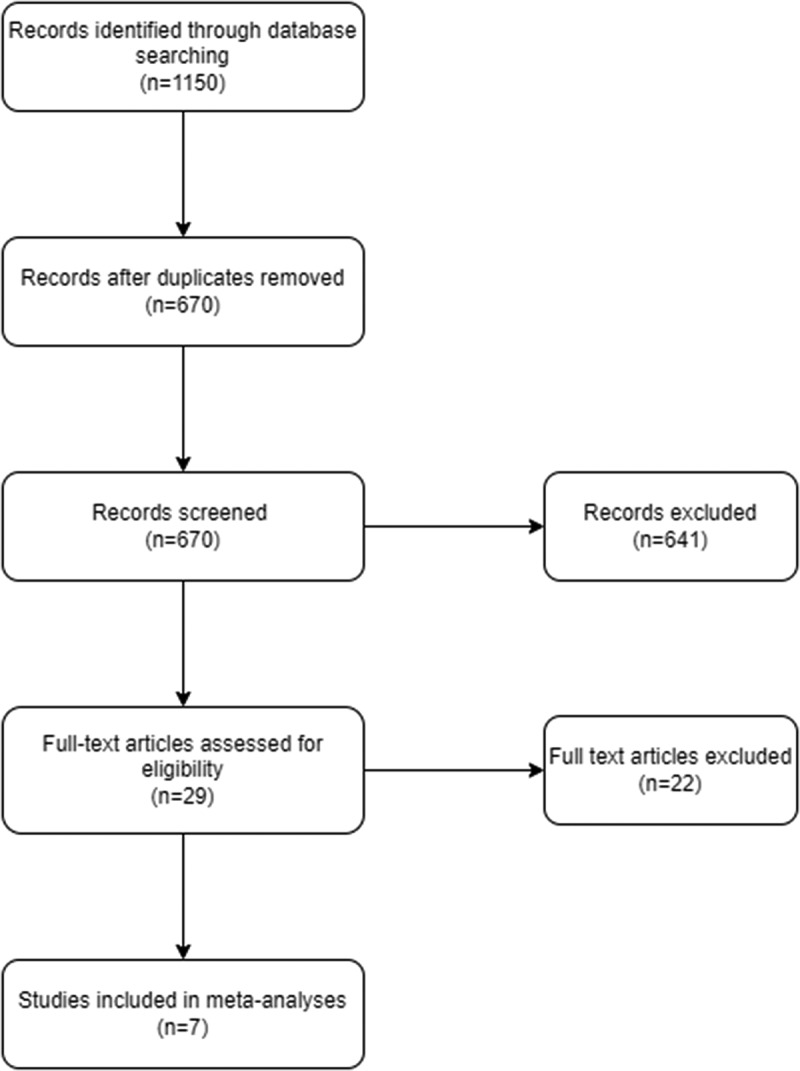
Flowchart of the study.

As a result of literature screening, 1150 records were obtained. After removal of duplicates, we screened the titles and abstracts of 670 records, excluded 641 and then screened 29 full-text articles from which we excluded 22 references, which did not meet our inclusion criteria; see PRISMA flowchart (Figure 1) for further details. The remaining 7 RCTs were included in the meta-analysis for qualitative and quantitative synthesis [19–25].

The study included a total of 373 urethral stricture patients in 7 RCTs. The studies were performed in Canada [1], the United Kingdom [1], Germany [1], India [2], Egypt [1], and Bangladesh [1]. The age range of participants was 17 to 72 years. Three studies included participants with either penile or bulbar urethral strictures [21,22,24], one study only included participants with bulbar strictures [23], and three studies made no reference to stricture location [19,20,25]. Three studies obtained graft from unilateral cheek [23–25], 2 studies used unilateral cheek in addition to bilateral cheek and lower lip for some patients [20,21], 1 study used lingual mucosa graft [22] and 1 study did not give information about the location of the graft [19]. The shape of the graft was rectangular in 2 studies [21,23], ovoid in 2 studies [24,25], and information about the shape of the harvested graft was not given in 3 studies [19,20,22].

In five studies, graft harvest was preceded by buccal infiltration with various local anesthetics [0.25% Marcaine with epinephrine [25]; 1.0% lidocaine with epinephrine [22,23]; 2% lidocaine with epinephrine [24];1% xylocaine and adrenaline [21]].

Closure techniques and suture choice varied between studies with 3 studies employing continuous closure [20,21,23,25] and 2 studies using interrupted suture [22,24], while 1 study did not state this information [19]. One study used a 5-point pain scale [21], while the other studies used 10- or 11–point pain scales for oral pain assessment [23–25], and 2 studies did not define the scale used. The detailed descriptions of the included RCTs are summarized in Table 1.

**Table 1.** Summary of randomized controlled studies characteristics.

Study	Country	Year	StudyDesign	Non-closure(no)	Closure(no)	Age, (mean) Non-closure / Closure(years)	İndication for BMG	Inclusion criteria	Excluding criteria’s	Stricture length(cm),(mean) Non-closure / Closure
Abdallah,2021	Egypt	2019-2020	RCT	15	15	37(18-63) / 35.5(17-64)	Urethral stricture	Patients with urethral stricture disease undergoing urethroplasty using buccal mucosal graft	Bleeding tendency, Oral pathology, and constraints of harvesting buccal mucosa	6.77 (2.7-12) / 4.42 (2.5-8.3)
Gulani,2019	İndia	2015-2016		21	21	37.7+-9.3 / 36.5 +- 9.1	Urethroplasty	No description	Oral cancer and ulcerSubmucosal fibrosis, Poor mouth openingPrevious oral surgery and Bilateral lingual harvest site	8.45+-1.1 / 8.63+-1.0
Soave,2017	Germany	2014-2015	RCT	72	63	53(34-66) / 55 (39-68)	Uretroplasty	Male patients > 18 yr of age with urethral stricture disease	previous treatment with BMGU, concomitant oral diseases Psychiatric disorders or cognitive impairment Chronic pain, bilateral buccal mucosa graft harvest Non-German speaking patients.	2.5(2-4) / 3(2-4)
Kabır, 2017	Bangladesh	2012-2014	RCT	16	16	41.3/42.4	Urethral stricture	All male patients with stricture urethra	Cognitive impairmentHistory of oral malignancyRefused to participate in the study	7.53 /4.75
Wong, 2014	United Kingdom	No description	RCT	18	16	41(23-59) / 47(31-64)	Urethroplasty	No description	Previous oral mucosa harvesting,Bilateral cheek, lingual, or lip oral mucosa harvests	No description
Rourke, 2012	Canada	2006-2008	RCT	24	26	44.4 / 43.8	Urethral reconstruction	bulbar urethral reconstruction with unilateral BMG harvest.	Bilateral BMG graft harvestingNon-English speaking patients Cognitive impairment, History of oral cancer	4.0 / 4.7
Muruganandam, 2009	İndia	2003-2005	RCT	25	25	35(18-64) / 35(17-72)	Uretroplasty	No description	No description	8.4+-2 (2-17) / 5+-1.5 (3-10)

Buccal source and harvesting graft shape	Graft origin	Graft length, (cm)(mean, range)Non-closure / Closure	Local anesthesia	Hemostasis approach	Closure technigue sutures	Surgical Endpoint’s	Endpoint’s assesment tool’s	Oral antiseptics	Follow-up
No description	Cheek Buccal mucosa	7.7 (3-14) / 5.2 (3-9)	No description	No description	No description	Pain score,Perioral numbnessDifficult mouth openingTolerate liguids and solidsSalivatory problems	Visual analog scala (VAS)	No description	Nodescription
Graft harvesting was done by standart technigue.	Lingual Mucosa	Graft width was same in both groups (1.5-2.2 cm)	With adrenaline (1:100.000)	bipolar cauteryadrenaline-soaked ribbon gauze	chromic catgut 3-0 suture	Pain score, Pain at the graft harvest siteDifficult tongue protrusion, Swallowing liguid diet Swelling of the graft harvest site,İncrease saliva productionEating soft and regular diet, Numbness of the graft harvest siteDysgeusia and Speech impairment	visual analog pain score (VAS) non-validated questionnaires	No description	No description
Graft harvested in a ovoid shape from the inner cheek	Cheek Buccal mucosa	4.5(4-6) / 5(5-8)	2% lidocaine with adrenaline	Bipolar electrocautery One piece of cottonoid gauze	interrupted 4-0 monofil sutures	intensity and quality of pain and oral morbidity (mouth opening,perception of taste, salivation, oral sensation, diet, oral bleeding, use of analgesics, smiling, whistling, oral swelling,speech and burden in daily life)	11-point NRSMc Gill Pain Questionnaire (SF-MPQ)non validated stage scala	Daily oral rinsing with chamomile	6 month
İn closure group, single cheek İn non closure group, both cheeks and lower lips	Cheek Buccal mucosa	N/A	N/A	Electrocautery adrenalin soaked gauge	continuous interlocking 3 ‘0’ vicryl sutures	Pain at BMG harvest site regular diet and difficulty in mouth opening	Visual analog score (VAS)Self made questionnaires	Chlorhexidine oral rinses	3 month
An ovoid shape with a width ranging from 2 to 2.5 cm,The lengths varied according to the stricturotomy.	Cheek Buccal mucosa	7.8+-1.5 / 8.8+1.5 (Graft area, cm2, mean+- SD)2 / 2 (Graft width, cm)	0.25 % Marcain with adrenaline (1:200.000)	Bipolar diathermyA wet gauze	4-0 Vicryl rapide sutures in a continuous fashion with knots buried.	pain, numbness, tightness, and the ability to drink and eat.	10-point visual analog scala (VAS)	0.2% chlorhexidinedigluconate	1 year
a rectangular fashion on the inner cheek size of 6 cm long and 2.5 cm wide.	Cheek Buccal mucosa	2.5 x 6 (all groups)	%1 lidocaine with epinephrine	Bipolar electrocautery,cottonoid gauze soaked in 1% lidocaine with epinephrine	running 3-0 chromic suture	the morbidity of the BMG harvest sitethe interval to a regular diet, changes in salivation, interval to full mouth opening, and the presence of perioral numbness.	10-point numeric pain scale(VAS)] non-validated questionnaire	N/A	6 month
the single cheek (15/25 vr 19/25; nonclosure vs closure).Six patients in nonclosure group had buccal graft harvested from the lower lip.	Cheek and lower lip buccal mucosa	The are of the harvested graft (cm2)(14.9 / 8.6)	The submucosal plane is infiltrated with 1% xylocaine and adrenalin solution (1 in 100.000)	bipolar electrocautery adrenalin soaked gauge piece	continuous interlocking sutures using 3 ‘0’ vicryl	assess pain, mouth opening and loss of sensation at graft site.	Self made questioners, Visual analog scale (VAS)	No description	No description

**Figure 2.**
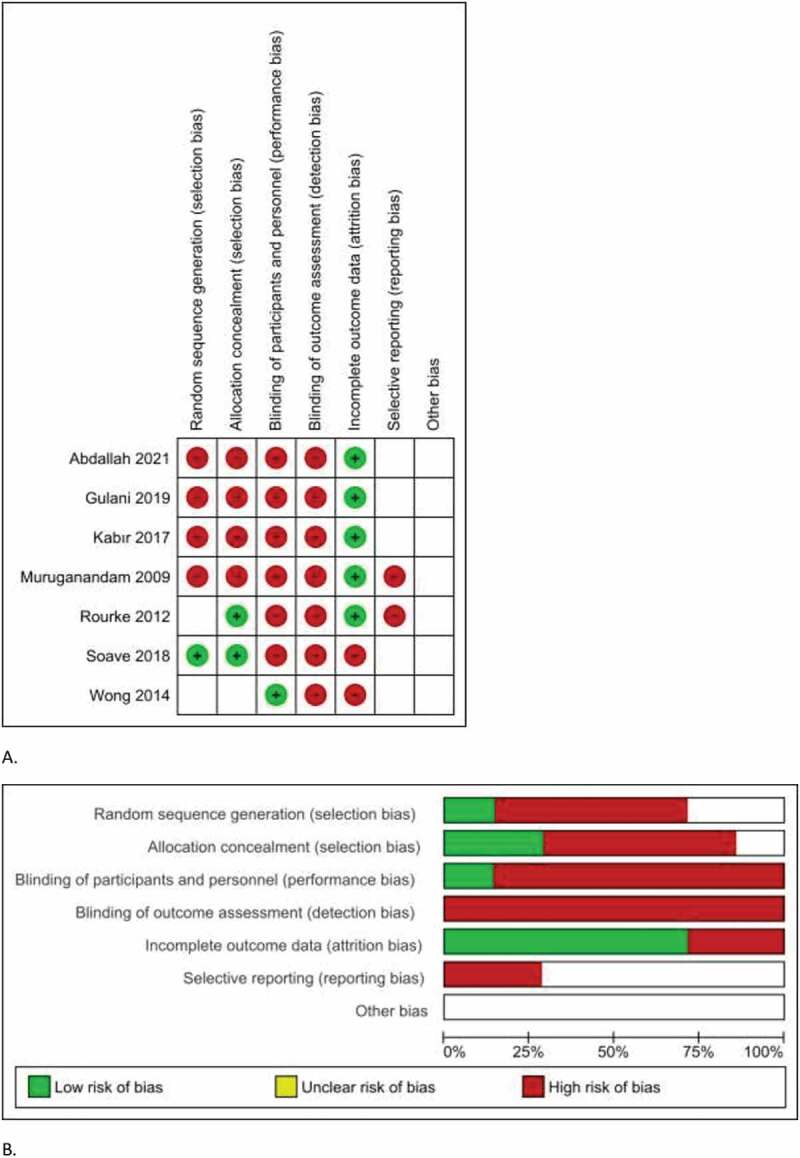
A.Risk of bias summary, B. Risk of bias graphy.

All included studies had an unclear or high risk of bias across several domains potentially susceptible to selection bias, performance bias, detection bias, and attrition bias. Regarding selection bias, four studies [19–22] used a quasi-randomization method. The separate risk of bias assessment for each study is summarized in Figure 2.

## Primary outcomes

### Difficult mouth opening

This was assessed in 4 subgroups of the postoperative 1st day, days 5–7, 1–3 months and 6 months. The total overall pooled effect estimates for 5 studies showed no statistically significant difference between the closure and non-closure groups (RR:0.89, 95% CI 0.76, 1.03, I^2^: 57%, p = 0.11).

Subgroup investigation observed that the non-closure group was statistically advantageous for difficult mouth opening in month 6 subgroups (RR:0.59, 95% CI 0.36,0.97, P = 0.04,I^2^:0%), with no statistically significant difference observed in the day 1,day 5–7 and month 1–3 subgroups.

**Figure 3.**
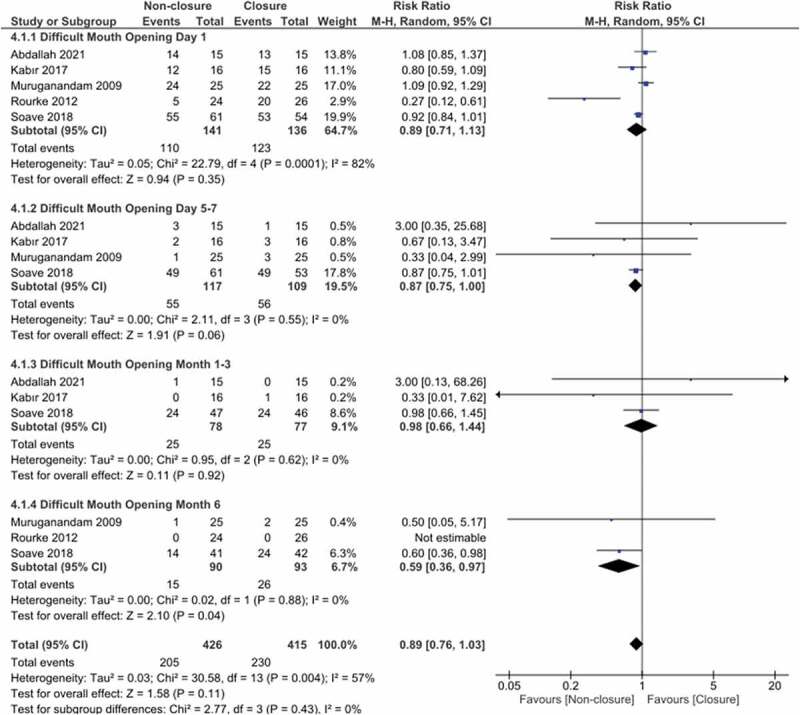
Forest plot pooled effect estimates for outcome of difficult mouth opening.

Heterogeneity was observed in the day 1 subgroup and the total group (Chi^2^ = 22.8, p < 0.001, I^2^:82%, Chi^2^: 30.6, p = 0.004, I^2^:57%). Repeated sensitivity analyses found heterogeneity was insignificant when the Rourke (2012) study was removed (Chi^2^:5.4, p = 0.14, I^2^:44% and Total; Chi^2^: 15.06, p = 0.24, I^2^:20%). After correction for heterogeneity, the overall pooled effect estimates for the subgroups and the total overall pooled effect estimates did not change(Figure 3).

### Perioral numbness

**Figure 4.**
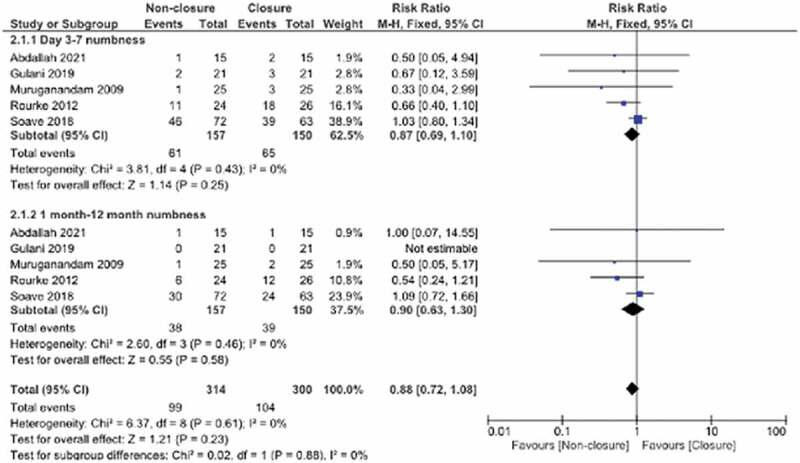
Forest plot pooled effect estimates for outcome of oral numbness.

Five studies examined perioral numbness. The day 3–7 and 12 month subgroups were assessed. The total overall pooled effect estimates did not identify a statistical difference in the non-closure and closure groups (Total RR:0.91, 95% CI: 0.76,1.08, p = 0.28, I^2^: 13%) (Figure 4).

### Pain score

**Figure 5.**
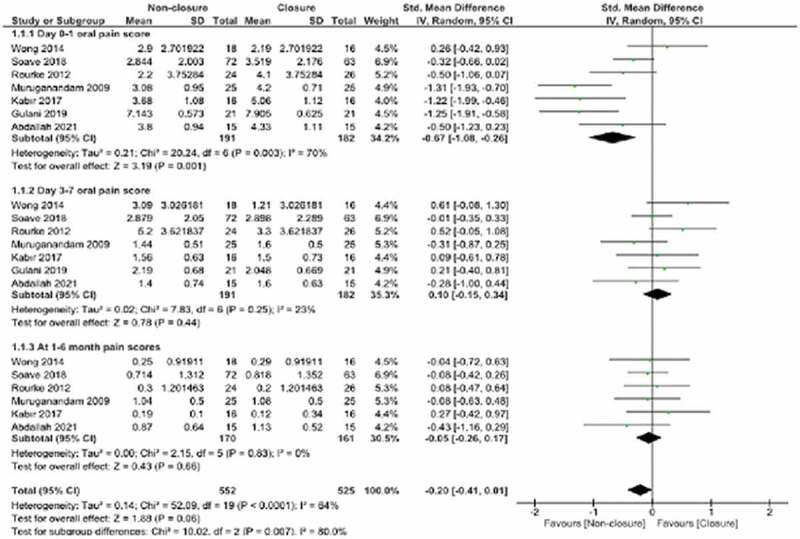
Forest plot pooled effect estimates for outcome of postoperative oral pain.

Pain was investigated in 3 subgroups (day 0–1, day 3–7 and month 1–6) and data were obtained from 7 studies. The total overall pooled effect estimates did not identify a statistically significant difference between the non-closure and closure groups (SMD:-0.20, 95% CI:-0.26,0.01, p = 0.006, I^2^: 64%). In the day 0–1 oral pain subgroup, the overall pooled estimates found the non-closure group was more advantageous (SMD: −0.67, 95% CI-1.08,-0.26, p = 0.001, I^2^ 70%). It appeared that heterogeneity was high in the day 0–1 subgroup and in total (Day 0–1 and Total; Chi^2^:20.24, p = 0.003, I^2^:70% and Chi^2^:52.1, p < 0.001, I^2^:64, respectively). Sensitivity analysis found the source of the heterogeneity were the studies by Muruganandam (2009) and Gulani (2019). When these studies were removed, the new assessment of the day 0–1 subgroup found that the heterogeneity was significantly improved (Chi^2^:8.47, p = 0.08, I^2^:53%). For the total oral pain score, heterogeneity was lost (Chi^2^:27.39, p = 0.05, I^2^:38%). As a result of this correction, the statistically significant difference in favor of the non-closure group did not change for the day 0–1 oral pain score subgroup between the non-closure and closure groups (SMD:-0.42, 95% CI: −0.80,-0.04, P = 0.03, I^2^:53%). Similarly, there was no variability in the total overall pooled effect estimates (SMD:-0.20,95% CI: −0.41, 0.01, P = 0.30) (Figure 5)

### Alteration of salivation

**Figure 6.**
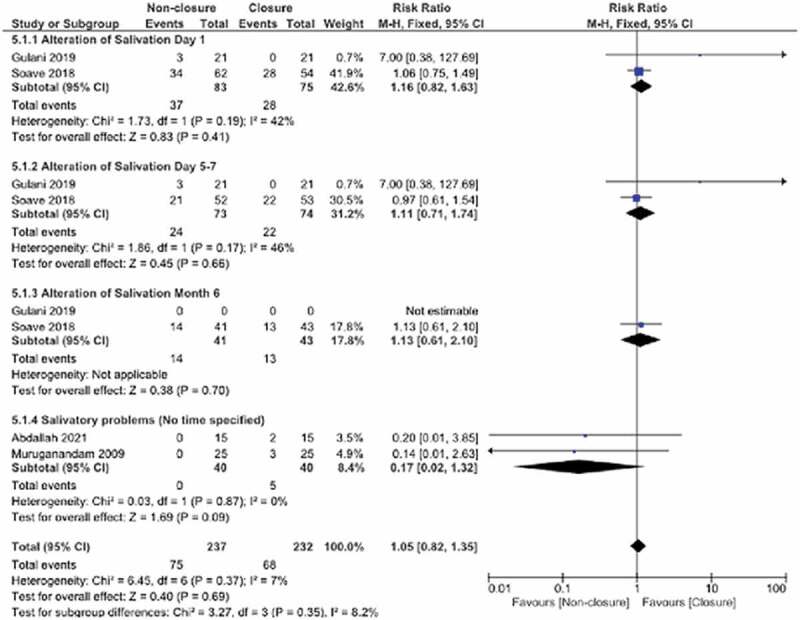
Forest plot pooled effect estimates for outcome of alteration of salivation.

Four studies were observed to investigate oral salivation. Considering studies presenting data at time intervals in the postoperative period, the subgroups for day 1, day 5–7, month 6 and no time specified were created. In terms of total overall effect estimate values, there was no statistically significant difference between the non-closure and closure groups (SMD: 1.05, 95% CI 0.61,2.10, p = 0.87, I^2^:7%). Significant differences were not observed in the subgroups (Figure 6).

### Toleration of liquid and solid intake

According to the total overall pooled effect estimates for tolerance of liquid and solid food, there was no statistically significant difference between the non-closure and closure groups (RR:0.96, 95% CI 0.90,1.02, P = 0.21, I^2^: 38%).

**Figure 7.**
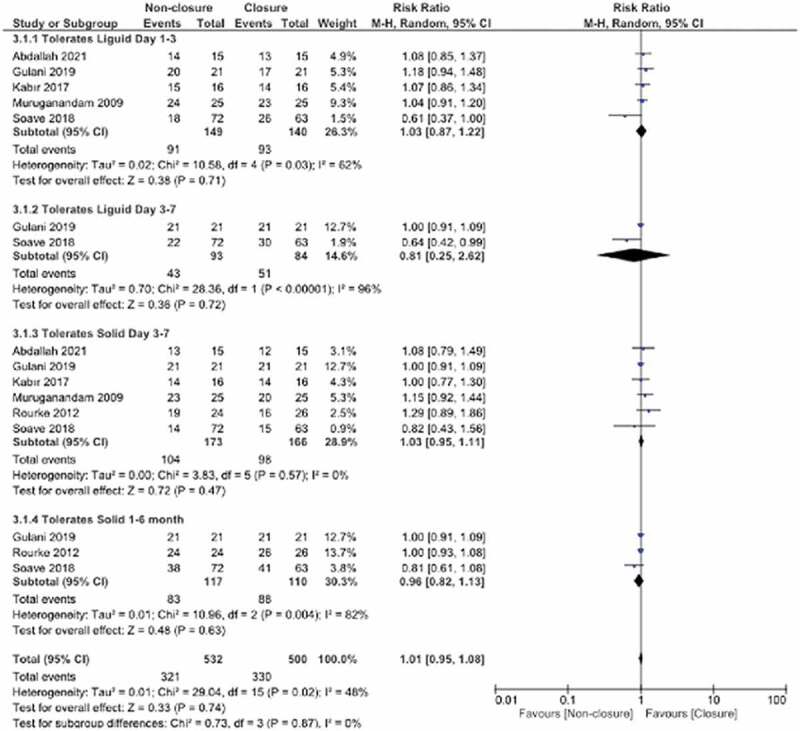
Forest plot pooled effect estimates for outcome for tolerates liquid and solid intake.

Liquid tolerance was considered at 2 time points. The day 1 time point was considered in 5 studies, while the day 3–7 time point was considered in 2 studies. These time point subgroups had similar pooled effect estimates between the non-closure and closure groups. For tolerance of solid food, two time point subgroups were obtained. Data for the day 3–7 subgroup were assessed in 6 studies, while data for the 1–6 month subgroup were assessed in 3 studies. In both subgroups, the pooled effect estimates were similar between the non-closure and closure groups (Figure 7).

## Publication bias

**Figure 8.**
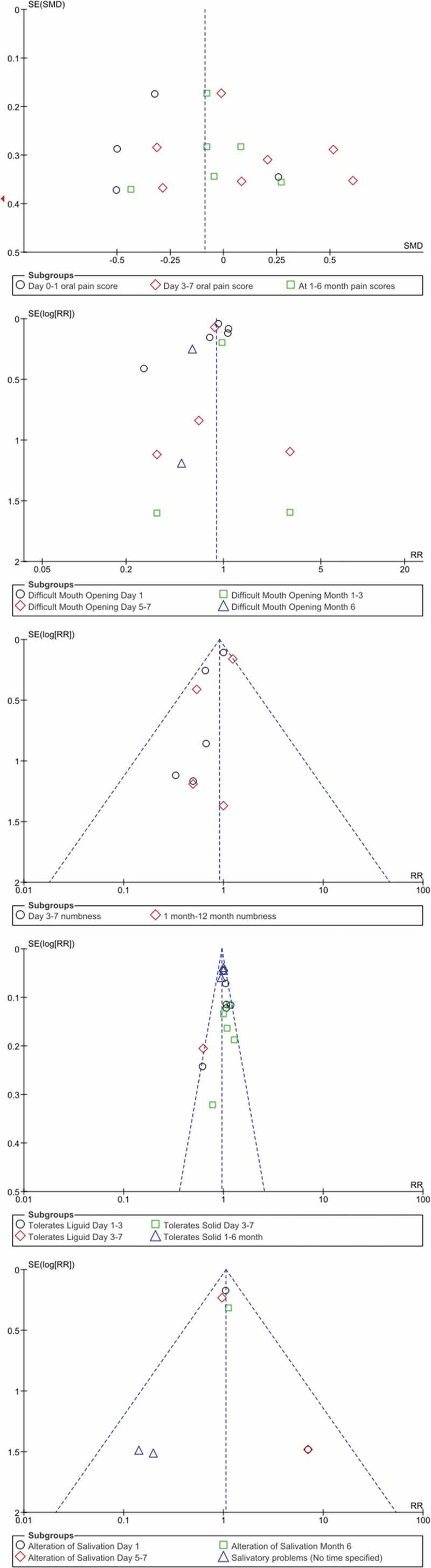
Funnel plot of effect sizes for clinical trials included in the meta-analysis A. Pain scores, B. Difficult mouth opening, C. Oral numbness, D.Tolerates diet, E. Alteration of salivation.

Potential publication bias was evaluated by identifying visual asymmetry/symmetry on funnel plot studies(Figure 8a-d). The graph that is not completely symmetrical indicated some publication bias. This is also one of the limitations of the present study.

### Discussion

In terms of postoperative oral pain, the overall effect estimates for the day 1 time point subgroup showed the non-closure group was statistically significantly superior to the closure group. This superiority was not observed at other time points and in the subgroups overall. Primary suturing of the donor cavity causes tension in the inner cheek due to approximation. Due to edema and inflammation continuing for mean 48–72 hours, patients in the closure group were identified to have higher pain scores, especially in the first postoperative days [26]. Contrary to this, some studies interestingly reported that pain increased between days 3–7 due to tissue contracture in the open donor field in the non-closure group. However, in this meta-analysis, we could not confirm this difference statistically. There are several studies recommending leaving the graft field open due to advantages in terms of postoperative pain [27,28]. However, oral pain reduces toward the end of the first week and is reported to be fully resolved by about 3 weeks [29].

Difficult mouth opening was similar in both groups at the day 1 time point due to probable edema and inflammation. For the day 5–7 time point, 3 out of 4 studies observed the number of patients with difficult mouth opening seriously reduced. This reduction continued for 1–3 months and the study by Soave et al., reporting high rates, showed a clear reduction in this rate. However, questioning in the 6th month in the study by Soave et al. observed that improvement continued in the non-closure group in parallel with time, while the closure group had similar rates to 1–3 months. As a result, the overall pooled effect estimates were very close to statistical significance in the day 5–7 subgroup and statistically significant in the month 6 subgroup; showed that the group that does non-close is more advantageous in terms of difficult mouth opening. However, it should be noted that this advantage is due only to the study of Soave et al., so we cannot generalize. It was reported that suturing of the donor field may cause difficult mouth opening in the future linked to scars and contraction [30], while some patients reported tightness in the mouth [29] and some patients reported persistent difficult mouth movements [27]. Contrary to this, there are studies reporting no patient had difficulty with mouth opening movements with suturing of the donor field and assessment in the 4th [13] and 6th months [26]. In a study by Barbagli et al [31]. suturing the unilateral cheek donor defect, only 1.7% of patients reported mild and moderate severity difficult mouth closure in the postoperative 3rd month. Patel et al [32]., in a study with non-closure of the buccal graft harvest field, concluded that preoperative mouth opening was approached in the postoperative 1st month and reached preoperative measurements in the 6th month. They reported that the size of the buccal mucosa harvest defect did not cause a difference in this situation.

Temporary oral paresthesia may occur due to neuropraxia of buccal and mental nerves after buccal graft harvest [31]. There are studies stating 57% of patients report development of oral numbness [29]. Five studies in this meta-analysis provided dichotomous data about postoperative oral numbness. For the time points and in total, statistical significance was not obtained for overall effect estimates for oral numbness complaints in the non-closure and closure groups. In 3 of the 5 studies including the time point of day 5–7, there were low numbers of patients with oral numbness, while the other 2 studies observed this complication at higher rates. It is notable that these 2 studies continued to have severe rates of this complication at the time point of months 1–12. Just as there are studies reporting 26% of patients experienced the oral numbness complication lasting 6 months or longer [27], there are studies reporting low numbers of patients with the numbness complaint during assessments at 3 weeks [26] and 4 months [31]. It is reported that this complication is more frequently observed for grafts taken from the lower lip [33,34].

Salivation changes and disorders like increased salivation, parotid sialadenitis and mucous retention cysts may be identified after buccal mucosa harvesting. In this meta-analysis, 4 domains were investigated of 3 time points and no time specified and no changes in oral salivation in the non-closure and closure groups were encountered. It is understood that the number of patients complaining about salivation changes reduces over time. To prevent changes to oral salivation in the postoperative period and salivary gland cyst and/or abscess, it is very important to identify and preserve the Stenson canal at the level of the 2nd molar tooth during graft harvest.

According to total pooled effect estimates for tolerance of solid and liquid diet, there were similarities between the non-closure and closure groups. One study observed liquid tolerance was very good from the postoperative 1st day. Assessment of tolerance of solid food on postoperative 3–7 days with data presented in 6 studies found very good tolerance of solid food, apart from one study. Barbagli et al [31]. reported that 58.6% of patients tolerated normal diet from the 3rd day in a prospective study suturing the graft defect after unilateral cheek ovoid donor harvest. Another study with a larger series reported 57% had solid food tolerance from the 3rd day. At the month 1–6 time point, it was understood that nearly all tolerance had fully improved.

The strengths of this meta-analyses include a comprehensive literature search for relevant comparative studies that assess the effects of non-closure vs. closure of the BMG harvest sites. Additionally, all studies included in the meta-analysis were RCTs and the adequate postoperative follow-up durations further strengthen this study. Barbagli [13] reported that suturing of the ovoid graft harvest site in the unilateral cheek increased patient satisfaction, while bilateral cheek graft harvesting was the only predictive factor for patient dissatisfaction. For this reason, the lack of investigation of subdomains related to age, sex, pediatric age group, shape of graft, area of graft (unilateral cheek/bilateral cheek and sublingual, lower lip), graft length, graft width, suture type, suture thickness, and factors disrupting oral hygiene are limitations of this study. Additionally, some RCT studies had high risk of bias and the presence of heterogeneity lowered the quality of this meta-analysis. There is a need for large participation, multicentric prospective studies examining homogeneous graft shape and graft dimension subgroups, including validated interrogation for assessment of oral complications apart from oral pain. Only in this way will it be possible to offer advice about making decisions about this topic by direct comparison of the non-closure and closure methods for healing of the BMG harvest site.

## Conclusion

As a result of this meta-analysis, leaving the BMG harvest site open may be recommended to reduce oral pain in the early postoperative period. There was no difference between the two groups in terms of oral numbness, alteration of salivation and consumption of liquid and solid food. Although the non-closed group seems more advantageous in terms of ease in mouth movements, more studies are needed to prove this.

## Supplementary Material

Supplemental Material
