# Supplementary figures and images for: Comparison of closure versus non-closure of the intraoral buccal mucosa graft site in urethroplasties. A systematic review and meta-analysis
Source: Arab J Urol. 2022 Jul 18;21(1):18–30. doi: 10.1080/2090598X.2022.2097613 (PMC9930765; doi:10.1080/2090598X.2022.2097613)

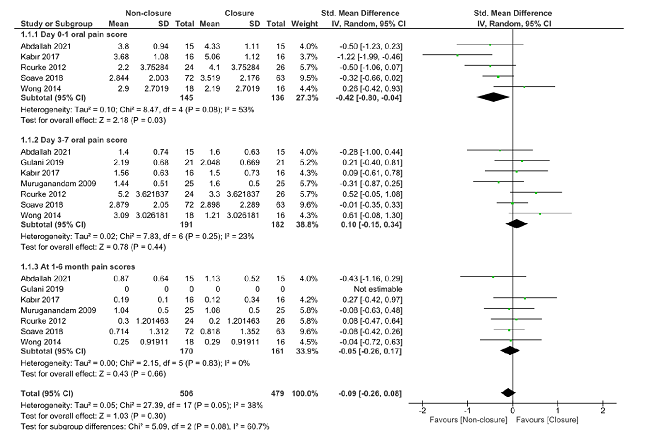

Supplement: Supplemental Material [file TAJU_A_2097613_SM7892.zip › suplemantary-figure-2-.png]

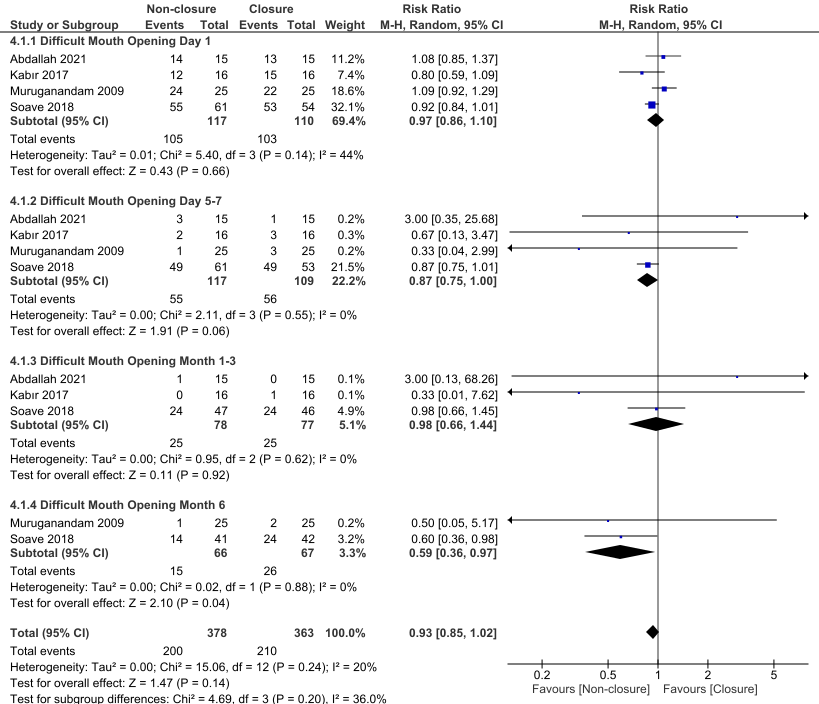

Supplement: Supplemental Material [file TAJU_A_2097613_SM7892.zip › Suplementary Figure 1.png]
